# Evolution of Surface Topography and Microstructure in Laser Polishing of Cold Work Steel 1.2379 (AISI D2) Using Quadratic, Top-Hat Shaped Intensity Distributions

**DOI:** 10.3390/ma15030769

**Published:** 2022-01-20

**Authors:** André Temmler, Magdalena Cortina, Ingo Ross, Moritz E. Küpper, Silja-Katharina Rittinghaus

**Affiliations:** 1 Fraunhofer Institute for Applied Optics and Precision Engineering (IOF), Albert-Einstein-Straße 7, 07745 Jena, Germany; 2 Fraunhofer Institute for Lasertechnology (ILT), Steinbachstraße 15, 52074 Aachen, Germany; magdalena.cortina@sms-group.com (M.C.); ingo.ross@rwth-aachen.de (I.R.); silja-katharina.rittinghaus@ilt.fraunhofer.de (S.-K.R.); 3 Lasertechnology, RWTH Aachen University, Steinbachstraße 15, 52074 Aachen, Germany; moritz.kuepper@llt.rwth-aachen.de

**Keywords:** laser remelting, surface roughness, laser polishing, micro hardness, microstructure

## Abstract

Within the scope of this study, basic experimental research was carried out on macro-laser polishing of tool steel 1.2379 (D2) using a square intensity distribution and continuous wave laser radiation. The influence of the individual process parameters on surface topography was analyzed by a systematic investigation of a wide range of process parameters for two different, square laser beam diameters. Contrary to a typical laser polishing approach, it was shown that short interaction times (high scanning velocity and small laser beam dimensions) are required to reduce both micro-roughness and meso-roughness. A significant reduction of surface roughness of approx. 46% was achieved from Ra_ini_ = 0.33 ± 0.026 µm to Ra_min_ = 0.163 ± 0.018 µm using a focused square laser beam with an edge length of *d_L,E_* = 100 µm at a scanning velocity of *v_scan_* = 200 mm/s, a laser power *P_L_* = 60 W and n = 2 passes. However, characteristic surface features occur during laser polishing and are a direct consequence of the laser polishing process. Martensite needles in the micro-roughness region, undercuts in the meso-roughness region, and surface waviness in the macro-roughness region can dominate different regions of the resulting surface roughness spectrum. In terms of mechanical properties, average surface hardness was determined by hundreds of nano-indentation measurements and was approx. 390 ± 21 HV0.1 and particularly homogeneous over the whole laser polished surface.

## 1. Introduction

Laser polishing of metallic surfaces is an emerging manufacturing process that aims to replace time consuming and cost intensive manual polishing operations. Since manual polishing may strongly depend on the skills and condition of the worker, laser polishing has the potential for full automation and is suitable for small and medium sized complexly shaped components. Therefore, it is not surprising that the scientific effort in the understanding the laser polishing process of metals has been steadily increasing over the past decade. Willenborg [1] was one of the first to introduce multi-step, areal laser polishing of metals and demonstrated that a surface roughness of approx. Ra = 0.2 µm was possible to achieve on tool steels. Lamikiz et al. [2] demonstrated the feasibility of areal laser polishing on stainless steel components build in a selective laser sintering process and achieved a surface roughness reduction of up to 80% to approx. Ra = 1.2–1.3 µm. The authors concluded from their experimental results that the rougher the original surface, the more effective the process [2]. The understanding of laser polishing using pulsed instead of continuous wave (cw) laser radiation, or for short laser micro polishing (LµP), was significantly influenced by Perry et al. [3], who introduced the critical wavelength and showed that the melt duration is a key factor in LµP. Bordatchev et al. [4] delivered a detailed overview for laser polishing of metals and systematically evaluated the achieved results in terms of surface roughness for broad variety of different metallic materials. In his PhD. thesis, Nüsser [5] not only provided an in-depth investigation on LµP of Ti6Al4V and tool steel H11, but also gave a detailed overview of the progress in LµP until approx. 2015. More recently, Temmler et al. [6] investigated LµP of stainless steel AISI 410 and discussed present developments in laser polishing. In addition, the authors gave a comprehensive overview of the known effects of process parameters on surface roughness for LµP of metals. The review articles by Deng et al. [7] and Krishnan and Fang [8] create a decent overview of the current state of energy beam processes for surface modification with special focus on surface roughness reduction such as energy beam or laser polishing. The most recent scientific publications focusing on laser polishing of steels, on modelling of continuous wave laser remelting/laser polishing process, on special approaches of laser polishing, and on an analysis of the microstructural properties of laser polished surface layers of steels were described by Temmler et al. [9]. Nonetheless, the specific research conducted for tool steel D2 and for non-circular intensity distributions in laser polishing will be specifically introduced.

The cold-working tool steel 1.2379 (EN X153CrMoV12, AISI D2) is a commonly used grade for deep drawing tools. Due to its high carbon and chromium content, a precipitation of M_7_C_3_ and M_23_C_6_ carbides within the tough martensitic matrix can be achieved by heat treatment. Therefore, the deep drawing tools provide a high resistance against abrasive and adhesive wear in conventional forming operations (Sing et al. [10]). Currently, the tool steel D2 is receiving special attention due to many scientific publications on laser-assisted material processing. Among others, the material was used as a reference material in a so-called DFG priority program SPP1676 of the German Research Association (DFG), in which the special focus lay on extensive and detailed investigations on laser processes for lubricant-free forming processes such as deep drawing [11,12,13,14], cold extrusion [15,16], cold massive forming [17], or rotary swaging [18,19]. Laser remelting / polishing was specifically investigated for surface modification of D2 in the context of lubricant free cold extrusion of aluminum [16]. Since laser polishing is a highly thermal process with extreme temperature gradients of up to 10^9^ K/s a strong interaction of radiation and material is typical for this process [20]. This interaction leads not only to a smoothing of the surface due to melting and resolidification but can also lead to all kind of different surface defects such as hot-cracks, holes, and deformation of the work pieces due to induced thermal stresses [21]. Therefore, the specific material composition in combination with the properties of the laser radiation used has a significant effect on the polishing result or the achievable minimal roughness. Although a small surface roughness in the range of 0.05–0.1 µm has already been achieved on some materials [9], the basic understanding of what roughness can be achieved on which material is incomplete. This is underlined by the fact that currently no coherent theoretical or numerical model exists that can predict the surface roughness after laser polishing with high reliability. Although such approaches exist, e.g., for LµP (Vadali et al. [22], Ma et al. [23], Chow et al. [24]) or laser polishing/remelting using continuous laser radiation (Ukar et al. [25], Richter et al. [26], Temmler and Pirch [27]), none of these models takes into account material-specific structure formation processes during or after laser remelting or laser polishing, as described for example by Nüsser et al. [21]. In particular, the effects of surface structure formation are presumably the reason that some residual roughness always remains on the surface, since competing effects can only be suppressed at mutual cost [21]. Furthermore, effects in case of surface machining, i.e., overlapping machining of individual remelting tracks, are not considered in any model. However, it is precisely this effect that can have a significant influence on the resulting roughness. Furthermore, solid phase transformations, e.g., the transformation in martensitic steels, are not considered in any model, but at least have a significant influence on the resulting micro-roughness. The material-specific effects are due to numerous different material properties and laser beam properties, which in their entirety can only be integrated into a complete, coherent model with extreme difficulty. In scientific and application-oriented practice, this leads to the fact that an essential part of the scientific work consists of explicitly experimentally testing the suitability of a pairing of laser beam source and material on the basis of a variation of key process parameters, thus systematically extending the empirical data basis for laser polishing.

Laser polishing of tool steel D2 using CO_2_ and high-power diode laser radiation was investigated by Ukar et al. [28]. The authors used comparatively slow scanning velocities, large laser beam diameters, and high laser powers to reduce surface roughness on a single track down to the order of approx. Ra = 0.36–0.86 µm along the center of the single track. However, large quantities of deposited energies could lead to hot cracking, the resulting surface roughness was just evaluated for single tracks instead of areal laser polishing, and surface roughness seemed still not suitable for any potential application such as die and mold making. Ross et al. [16] demonstrated that in the laser remelting process chromium carbides were dissolved in the molten material, which resulted in a chemically more homogeneous surface boundary layer without macroscale segregations. Spranger and Hilgenberg [29] found analogously that pulsed laser remelting of AISI D2 leads to a significant dissolution of contained hard primary and secondary carbides. Spranger at al. [30] observed that a fine-grained microstructure was developed in the heat-effected zone, and concluded that it resulted from a temperature increase above austenitizing temperature and a subsequent self-quenching due to rapid cooling. In this context, Telasang et al. [31] hypothesized that a possible diffusion of carbon from supersaturated martensite might have led to larger fractions of carbides in the heat affected zone of tool steel H13. Additionally, Ross et al. [16] found that the use of a powder metallurgical remelted variant of 1.2379 seems more suitable for laser polishing since chromium carbides are generally smaller, dominantly spherically formed and homogenously distributed within the bulk material. Furthermore, the authors found that alloying elements such as Manganese and Aluminum are typically enriched at the top surface layer after laser remelting. This is in good agreement with Temmler et al. [9], who found a significant redistribution of alloying elements after laser remelting of tool steel H11.

Furthermore, the intensity distribution or specific shaping of laser beams become increasingly important for laser-based processes. Kaplan [32] for example discussed the influence of the laser beam profile in laser welding. More recently, Rasch et al. [33] investigated shaped laser beam profiles for heat conduction welding of aluminum-copper alloys. For joining of thin foils, Funck et al. [34] found that specifically ring-shaped intensity distribution are advantageous for laser processing. Völl et al. [35] pointed out that intensity distributions should be tailored to the specific requirements of an application and material(s) involved to induce an adapted (time-dependent) temperature profile. One specific, practical approach was demonstrated by Pütsch et al. [36], who achieved a highly dynamic laser beam shaping using a multi-segmented, membrane deformable mirror. Using a more classical optical set-up, including a shaped aperture that is projected onto the materials surface and used for processing, Kumstel and Lüken [37] demonstrated that non-circular intensity distributions are beneficial to reduce bulging of the remelted tracks.

Specifically addressing the use of square intensity distributions, Khare et al. [38] found for surface laser remelting that the use of a square intensity distribution should facilitate processing at higher speed with reduced risk of so-called centerline solidification cracking. According to Khare et al. [38], the development of melt pools shaped similar to a tear drop as it is characteristic for high scanning velocities, increases the chance of solidification cracking at the centerline of the remelted track due to a potential accumulation of segregation elements. Due to a change in the geometry of the solidification front resulting from a square intensity distribution the direction of microstructural growth is affected. This leads particularly to a more pronounced region of axial instead of columnar grain growth (Kou [39]).

This work is integrated into the field of laser polishing of steels. This study investigates surface structure formation, microstructure, and surface roughness evolution in laser polishing of AISI D2 samples starting at an initial surface roughness of Ra = 0.33 (+/−0.026) µm on a powder metallurgical remelted bulk material. The aim of this study is to decrease surface roughness of 1.2379+ by polishing using continuous wave laser radiation and to determine the resulting microstructure and corresponding surface hardness.

## 2. Materials and Methods

### 2.1. Opto-Mechanical Set-Up

**Figure 1:**
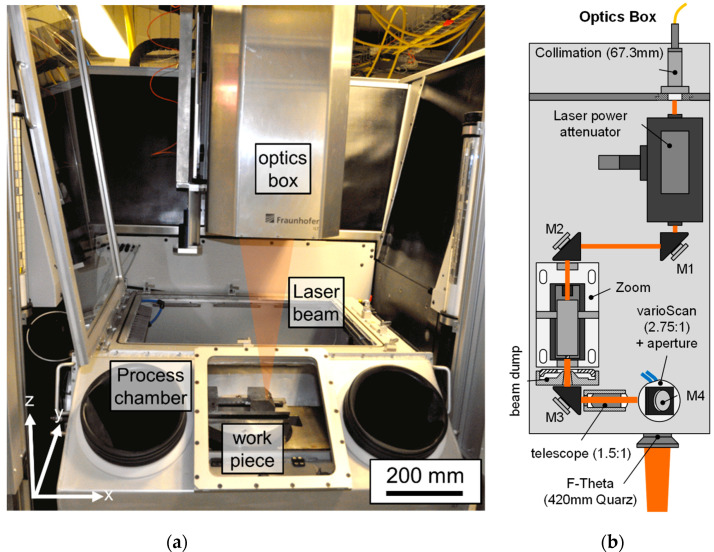
(**a**) Photo of processing chamber and optics box of the POLAR machine, and (**b**) schematic of the optical-setup and beam path [40].

As part of a publicly funded project, Fraunhofer Institute for Laser Technology (ILT) in cooperation with Maschinenfabrik Arnold (Ravensburg, Germany) developed the “POLAR” prototype laser polishing machine. A milling processing center C600U from Hermle (Gosheim, Germany) was used as mechanical basis for the POLAR system (Figure 1a). In order to create sufficient space for the integration of all components required for laser polishing, the milling spindle was removed from the headstock and instead parts of the optical system were integrated into it.

A diode-pumped Yb:YAG disk laser is used as laser beam sources for the POLAR machine (TruDisk 1000, Trumpf GmbH, Ditzingen, Germany). The TruDisk 1000 can only be used in continuous wave mode. The maximum average laser power is P_L,max,TD_ = 1000 W at an emitted wavelength of λ_em_ = 1030 nm. The laser radiation is directed into an optical setup (Figure 1b) via an optical fiber with a square fiber core and edge lengths of d_fiber_ = 100 µm. For further information, a detailed description of the optical set-up was recently given by Temmler et al. [40]. Focused laser beam diameters from approx. *d_L_* = 100 μm up to 800 μm can be used in this setup. Overall, five mechanical stages plus three “optical axes” enable the laser processing of components with sizes up to ∅ 300 × 250 mm^2^ and a maximum weight of approx. 200 kg. The laser processing takes place in a process gas chamber to avoid unwanted oxidation. The residual oxygen is monitored and adjusted by a closed-loop control.

### 2.2. Intensity Distribution and Laser Beam Source Orientation

For all experimental investigations, a fiber with a square 100 × 100 µm^2^ step-index fiber core was used for laser beam delivery to the optical system. The laser beam characteristics were systematically determined at two zoom positions adequate to achieve the desired laser beam dimensions in the focal plane. A MicroSpotMonitor (MSM) and an analysis software LaserDiagnoseSoftware v2.98 (LDS) both provided by Primes GmbH (Pfungstadt, Germany) were used for analyzing the caustic of the laser beam and the intensity distribution in and close to the focal plane.

**Figure 2:**
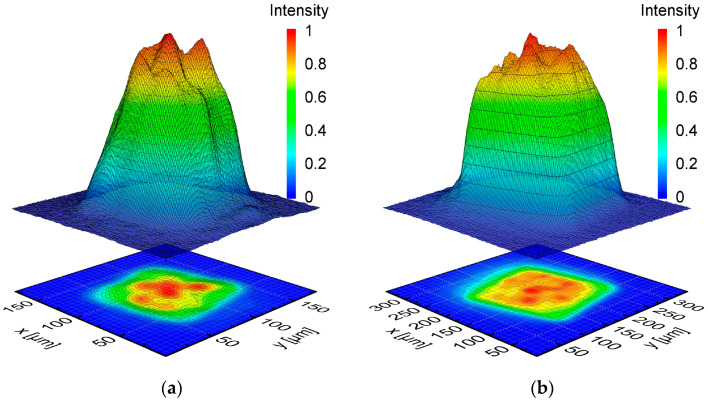
Normalized intensity distribution of square laser beams in their focal plane with (**a**) side lengths of 100 µm (Q100), and with (**b**) side length of 200 µm (Q200).

Figure 2 shows the measured intensity distributions for two square laser beam foci with the side lengths of 100 µm and 200 µm, respectively. In the following these will be referred to as Q100 and Q200. Based on 86% energy inclusion, the LDS analysis delivers the following laser beam characteristics for Q100 and Q200. For Q100, the beam quality *M*^2^ was analyzed to be *M*^2^ = 5.9, the Rayleigh length was *z_R_* = 3.16 mm, and the divergence angle was *Φ* = 50.27 mrad. The characteristics for Q200 were analyzed to be *M*^2^ = 9.31, *z_R_* = 4.15 mm, and *Φ* = 55.124 mrad. However, since LDS was not built to analyze square intensity distributions, some further analysis was necessary to reliably determine the geometrical dimensions of the laser beam. For this purpose, a square asymmetric super Gaussian distribution was fitted to the measured intensity distribution (Equation (1)) [41].

(1) $$I\left(x,y\right)=I_{0}\cdot e^{-2{\left({\left(\frac{x}{a}\right)}^{M}+{\left(\frac{y}{a}\right)}^{M}\right)}^{N}}=I_{0}\cdot e^{-2{\left({\left({\left(\frac{x}{a}\right)}^{M}+{\left(\frac{y}{a}\right)}^{M}\right)}^{\frac{1}{M}}\right)}^{NM}}$$

The square intensity distribution is centered at the origin and aligned parallel to the axes of a Cartesian coordinate system. The main characteristics are the beam diameter *a* and the numerical parameters *M* and *N·M*. An increasing *M* represents a higher degree of similarity to a square intensity distribution, while an increasing product of *N·M* indicates a higher similarity to a top-hat intensity distribution. The corresponding *M* and *N* for Q200 were calculated to be *M* = 6.89 and *N·M* = 6.93 and for Q100 *M* = 4.5 and *N·M* = 4.61.

### 2.3. Material and Sample Preparation

The cold work steel D2 (EN X153CrMoV12, 1.2379) is widely used in the field of cutting and punching tools as well as in solid forming. AISI D2 is a 12% ledeburitic chromium steel, which combines toughness, very high wear resistance, compressive strength, and dimensional stability. Particularly for the cold extrusion of aluminum, D2 is recommended as a material for tool dies, punches and ejectors, among other things [13]. A powder metallurgically produced material D2 (PM) from Dörrenberg Edelstahl GmbH (Engelskirchen, Germany), which is identical in its elementary composition to D2, was used in this investigation. This material is particularly characterized by a segregation-free microstructure, a high homogeneity in the elemental distribution, and a low content of impurities such as sulfur and phosphorus. The chemical composition (wt. %) of the material is summarized in Table 1.

**Figure 3:**
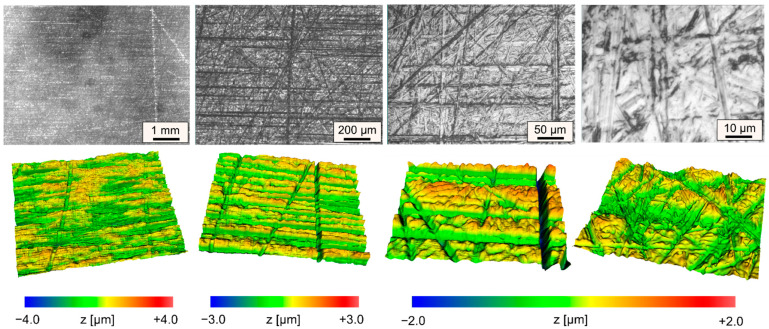
Initial surface topography in increasing magnifications; top row: microscopic images; bottom row: corresponding WLI measurements (same scale).

The initial heat treatment state of the samples is soft annealed, while the dimensions are approx. 50 × 75 × 15 mm³ for all samples. The initial roughness of the ground sample surface was Ra_ini_ = 0.33 ± 0.026 µm (Figure 3).

The experiments were carried out on flat samples using the previously described tools Q100 and Q200. For both tools, the laser spot size, track offset *dy*, and residual oxygen *c*(*O*_2_) in the chamber stayed fixed. Regarding laser polishing, a part of the surface always remained unaltered to enable characterization of the initial surface topography.

### 2.4. Process Principle, Scan Strategy, Process Parameters and Design of Experiments

**Figure 4:**
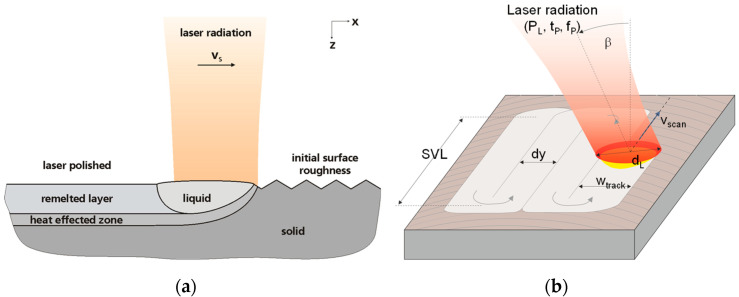
(**a**) Schematic of laser polishing process principle, and (**b**) schematic of process strategy and of process parameters typically relevant in laser polishing [40].

Laser polishing is similar to a micro-welding process without any additional material. The laser beam impinges on the surface of a material and creates a melt pool (Figure 4a). Capillary forces within the melt pool led to a smooth surface of the molten material. The smooth melt pool surface solidifies when the laser beam is moved over the surface and typically results in a significant reduction of surface roughness. A considerable overlap of adjacent remelt tracks leads to areal laser polishing (Figure 4b).

Macro polishing utilizes continuous wave laser radiation and was performed using the laser beam source TruDisk 1000. Two similar investigations were conducted for the laser beam tools Q100 and Q200 and compared among each other. The central, experimental approach was an investigation on laser power in eight equidistant discrete steps in order to find the minimum surface roughness for a given set of process parameters. In addition to two different laser beam diameters, the investigation on laser power was conducted for a matrix of three different scanning velocities and three different number of polishing passes in a full factorial approach. Overall, this study is based on laser polished test fields for 138 different sets of process parameters. An overview, a short description of the investigated process parameters and the range of investigation is given in Table 2. However, only representative results will be shown and discussed in this manuscript.

The selected track offsets *dy* correspond to a track overlap of 80% laser beam diameter and the minimum laser power was defined by the melting threshold, whereas plasma formation or visible evaporation of material was used for determining the maximum laser power. In this case, the maximum laser power *P_L_* for the 100 × 100 µm^2^ tool was limited to 80 W due to restrictions related to the machine’s configuration. Macro polishing investigations were analyzed attending to the employed number of passes n (1, 2, 4 passes) and scan velocities *v_scan_* (50, 100, 200 mm/s). Then, both Ra stylus measurement and Sa-spectra in combination with microscope (Leica, Wetzlar, Germany) and WLI images are presented and discussed.

### 2.5. Surface Analysis

For the evaluation of surfaces, these are often measured by means of stylus methods or white light interferometry and the average roughness along a line (Ra) or for an area (Sa) is determined. For a more detailed analysis of the roughness, the spectral composition of the roughness was analyzed and displayed in a roughness spectrum (Sa-spectrum) [1,5,6,9]. These Sa-spectra are based on WLI measurements, for which a “Newview 7300 Optical Surface Profiler” by Zygo Corp. (Middlefield, CT, USA) was used. The vertical resolution is max. 0.1 nm, whereas the spatial resolution depends on the objective and ranges from 0.36 to 9.50 μm. Magnification and zoom parameters among others were set by means of the provided control and analysis software MetroPro (V10.3) from Zygo Corp.

For a tactile roughness evaluation of the surface a M2 perthometer by Mahr GmbH (Göttingen, Germany) was used. This instrument serves for determining two-dimensional roughness parameters by moving a diamond head stylus horizontally over the area of interest. Thus, the vertical movement of the stylus determines the topography of the surface. The average roughness Ra was measured in accordance with DIN EN ISO 4287 for determining the tactile roughness.

### 2.6. Microstrutural and Micro-Hardness Analysis

Based on SEM images, a microstructural analysis was performed for selected sets of process parameters. Two extrema of the range of process parameters (Table 2) were investigated for single track remelting and areal laser polishing: small laser beam diameter (Q100) and high scan velocity (200 mm/s) as well as large laser beam diameter (Q200) and small scan velocity (50 mm/s) were chosen, each at their respective laser polishing power (laser power that led to a minimal surface roughness; c.f. Section 3.2). The corresponding sets of process parameters are shown in Table 3. After laser remelting/polishing single tracks and laser polished areas were cut perpendicular to the scanning direction of the last crossing. The corresponding cross-sections were metallographically prepared and etched with Vilella’s solution to visualize the corresponding microstructure.

The influence of process parameters for laser polishing on micro-hardness (Vickers hardness) was exemplarily investigated for the set of process parameters C and D displayed in Table 3. In this case, the micro-hardness HV0.1 (20 s) was investigated by means of nano-indentation (Picodentor HM500, Helmut Fischer GmbH, Sindelfingen, Germany) on the surface. Based on three different laser tracks and laser polished areas, respectively, 100 micro-hardness measurements were conducted in a lateral distance of approx. 20 µm and at an angle of approx. 10° (±2°) relative to the scan direction.

## 3. Results

### 3.1. Characteristic Surface Features

**Figure 5:**
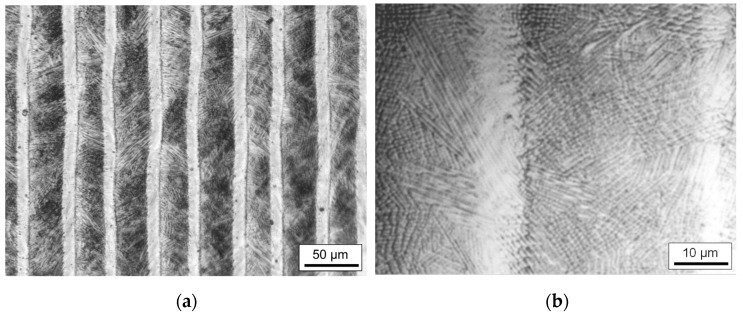
Micrographs of remelted surface at (**a**) low magnification, and at (**b**) higher magnification (Q200, *v_scan_* = 50 mm/s, *dy* = 40 µm).

Due to a meandering machining strategy, a characteristic stripe-like surface pattern was created for almost all laser polished test fields (Figure 5a). Darker parallel stripes alternate with lighter stripes. Depending on the process parameters used, the ratio of the respective stripe widths differs to each other. The bright stripes are particularly pronounced for a large laser beam and small scanning velocity.

**Figure 6:**
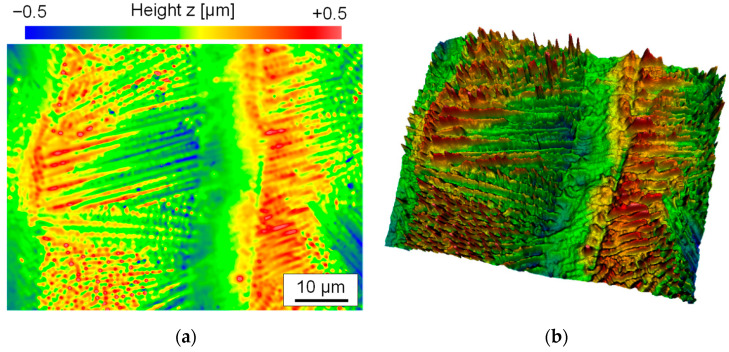
(**a**) 2D-WLI image and (**b**) 3D WLI image of the same surface topography after laser remelting with process parameters leading to a particularly high micro roughness (Q200, *v_scan_* = 50 mm/s, *dy* = 40 µm, *P_L_* = 80 W, n = 4).

A magnified section shows that the surface topography in the darker areas differs from that in the lighter areas (Figure 5b). The surface in the lighter areas appears much smoother than in the darker areas. This is most obvious for the largest laser beam diameter used, Q200, and the smallest scanning velocity of *v_scan_* = 50 mm/s. The surface topography in the micrometer range (micro-roughness) is dominated by martensite needles, which are formed during the remelting process due to the rapid solidification rates and the high carbon content dissolved in the iron matrix (Figure 6a). In comparison, the structures in the lighter areas are clearly smoothed, which explains the different visual perception of these surface areas (Figure 6b).

Presumably, the lighter stripes are not exclusively melted and re-solidified material, but this area was additionally heat-affected by the subsequently remelted track.

**Figure 7:**
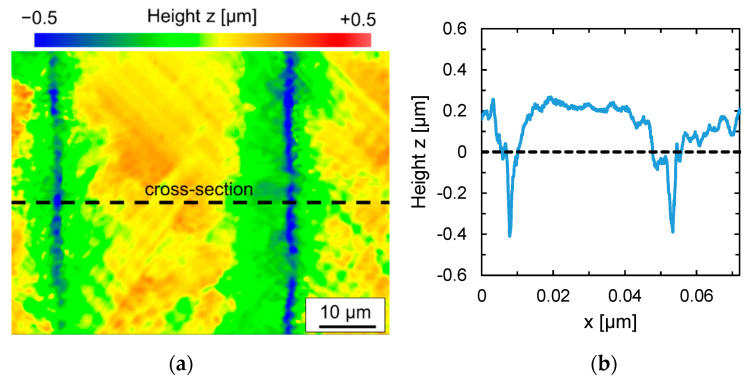
(**a**) WLI image of laser remelted surface and (**b**) profile of along marked cross-section (Q200, *v_scan_* = 200 mm/s, *dy* = 40 µm, *P_L_* = 110 W, n = 2).

When the scanning velocity is increased, not only does the formation of martensite decrease, but so-called undercuts appear to the left and right of a remelted track. As a result of subsequent partial remelting of a remelted track by the next adjacent track, these undercuts remain at the edge of each remelt track at the distance of the track offset. (Figure 7a). At the same time, these undercuts also mark the boundary between the darker and lighter areas of the striped pattern. The undercuts have depths of approx. 0.1 mm to 0.5 mm (Figure 7b) and occur particularly at high scanning velocities.

In addition to undercuts, the remelted tracks also show some significant bulging of a few tenths of micrometers (Figure 7), which might also increase the resulting surface roughness. Typically, this track bulging is a consequence of a microstructural transformation before and after laser remelting. Particularly, the formation of martensite is often related to an increase in volume.

**Figure 8:**
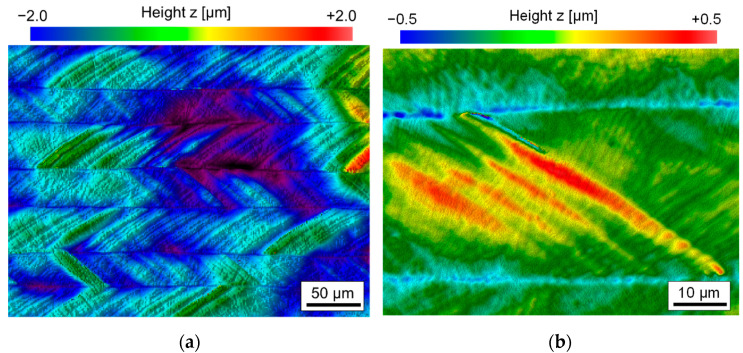
(**a**) WLI image of laser remelted surface and (**b**) magnification of ripple formation on laser remelted surface (Q200, *v_scan_* = 200 mm/s, *dy* = 40 µm, *P_L_* = 110 W, n = 2).

Furthermore, a large number of macro ripples were formed during the first pass of laser polishing. Due to the overlapping of single tracks only fragments of these macro-ripples remain on the remelted surface. These remnants of the macro-ripples are, however, omnipresent on a remelted surface (Figure 8a) after n = 1 pass. These fragments of macro-ripples were found on all laser polished surfaces and typically dominated the micro-roughness after the first pass (Figure 8b). Therefore, it can be assumed that they are directly connected to the chemical composition of the material in its initial state. Consequently, multi-stage processing was required in all cases to reduce or even eliminate the occurrence of macro-ripples. The rippling effect was most pronounced for the highest scanning velocities and moderate laser power.

### 3.2. Evolution of Surface Topography and Determination of Laser Polishing Power

**Figure 9:**
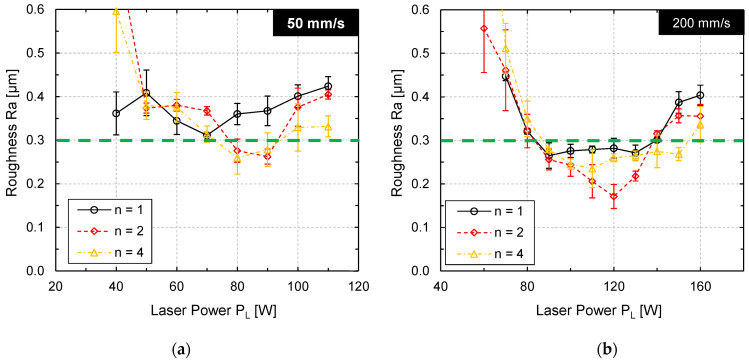
Surface roughness Ra (measured by tactile stylus method) as function of laser power for (**a**) *v_scan_* = 50 mm/s and (**b**) *v_scan_* = 200 mm/s after n = 1, 2, and 4 passes.

An effective way to determine the laser power most suitable for laser polishing is a systematic variation of laser power at otherwise fixed process parameters. The process limits are typically the laser power at which melting of the material starts and the laser power at which significant material evaporation occurs. After laser polishing at different laser powers, the surface topography was measured by stylus roughness measurements to determine the average surface roughness *Ra* (Figure 9).

Figure 9a shows that a local surface roughness minimum was achieved at laser powers of approx. *P_L_* = 80–90 W (*v_scan_* = 50 mm/s) after two and four passes, respectively. However, the surface roughness after laser polishing was not significantly reduced in comparison to the initial surface roughness (Figure 9, green dashed line). Figure 9b shows a similar result for a scanning velocity of *v_scan_* = 200 mm/s. The minimal roughness was achieved at approx. *P_L_* = 120 W for each number of passes n = 1, 2, and 4. However, only after two passes a surface roughness was measured that is significantly smaller (*Ra* = 0.175 µm) than the initial roughness (*Ra* = 0.33 +/− 0.026 µm).

**Figure 10:**
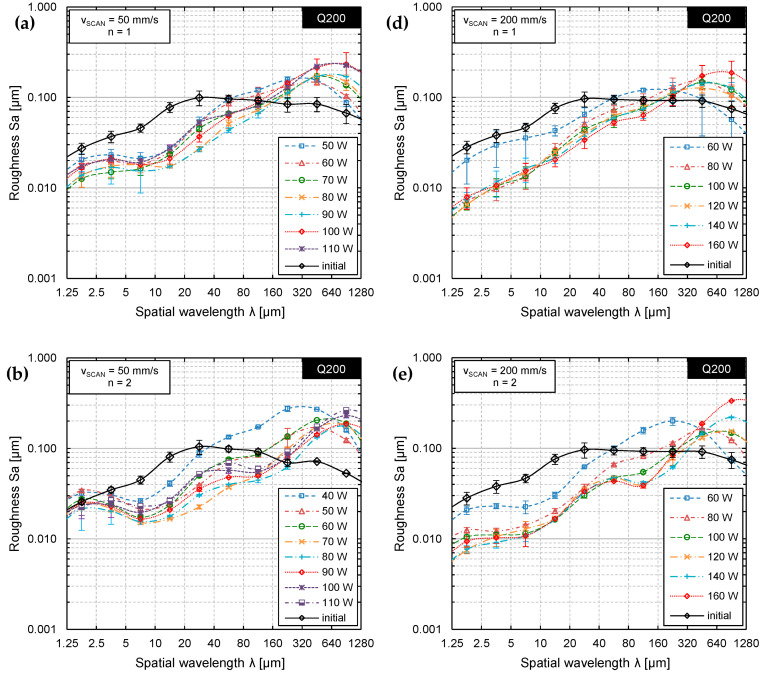

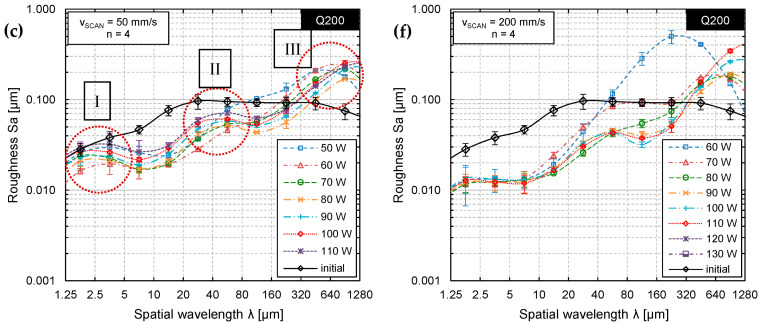
Roughness spectrum for Q200, *v_scan_* = 50 mm/s after (**a**) one pass, (**b**) two passes, and (**c**) four passes in direct comparison to Q200, *v_scan_* = 200 mm/s after (**d**) one pass, (**e**) two passes, and (**f**) four passes for different laser power *P_L_* from 50 W to 110 W and 60 W to 160 W, respectively.

This is not a typical evolution of surface roughness. Particularly, that surface roughness could only be reduced by one specific set of parameters is rather surprising. Therefore, the surface topography was measured by WLI and analyzed by Sa-spectra (Figure 10) to enable a more sophisticated analysis of surface roughness.

Figure 10a,b show that laser remelting has significantly different effects on the surface topography depending on the specific wavelength interval in the Sa spectrum. A comparison of Figure 10a,b shows that the resulting surface topographies seem to be very similar and indicate that no significant decrease of surface roughness was achieved by four laser polishing steps in comparison to one. Particularly, in Figure 10b, three different, characteristic wavelength intervals were identified in which only a small reduction of surface roughness or even an increase in surface roughness (waviness) was observed. In general, surface roughness was reduced up to a spatial wavelength of approx. 80 µm. In contrast to many other steels (materials), surface roughness at spatial wavelengths λ > 80 µm (macro roughness, region III) was not only not decreased, but significantly increased for all laser powers investigated. Typically, if the number of passes is increased, the surface roughness is further decreased. Figure 10b shows that this is not necessarily the case for tool steel D2. On the contrary, in comparison to the surface roughness spectrum after n = 1 pass (Figure 10a) an increase in two other specific wavelength intervals was observed. Firstly, the micro-roughness (λ < 5 µm, region I) was increased almost to a level of the initial surface roughness. Secondly, additionally the meso-roughness was increased (10 µm < λ < 100 µm, region II), if the number of passes increased.

Specific surface features that were induced by the laser remelting process were introduced and identified in the earlier chapter. These process-inherent or process induced surface structures can be associated to specific regions (wavelength intervals) in the roughness spectra. An increase of micro roughness (region I) might occur due to microstructural changes such as in martensite formation or process-inherent formation of surface structures such as ripples. A decrease in micro-roughness is typically achieved due to remelting of initial surface features such as milling grooves, grinding marks or surface scratches. In region II, small to medium spatial wavelengths were specifically affected by single track bulging and the formation of undercuts. In the third (III) region formation of waviness was observed, which had spatial wavelengths significantly larger than the laser beam dimensions. Typically, this kind of waviness cannot be significantly reduced by laser polishing, so that inducing this additional waviness should be avoided. This characteristic waviness was created by the remelting process and was more pronounced for high laser powers and a higher number of passes.

**Figure 11:**
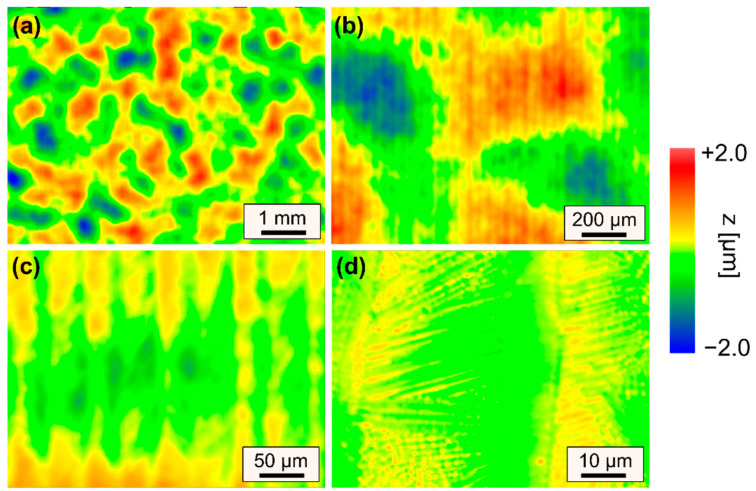
WLI measurements of surface topography for increasing magnifications (**a**–**d**) after laser remelting using Q200 and *v_scan_* = 50 mm/s leading to smallest surface roughness. (Q200, *v_scan_* = 50 mm/s, *dy* = 40 µm, *P_L_* = 80 W, n = 4).

**Figure 12:**
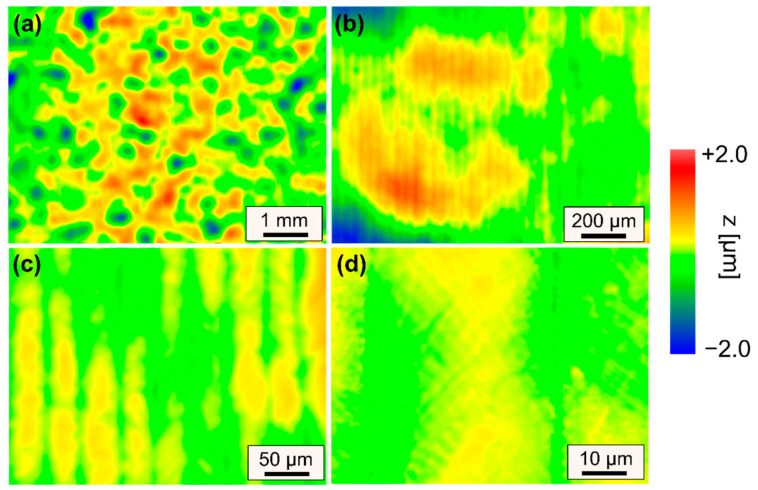
WLI measurements of surface topography for increasing magnifications (**a**–**d**) after laser remelting using Q200 and *v_scan_* = 200 mm/s leading to smallest surface roughness (Q200, *v_scan_* = 200 mm/s, *dy* = 40 µm, *P_L_* = 120 W, n = 2).

Representative surface features that lead to an increase in surface roughness are described based on WLI measurements of selected surfaces for specifically chosen sets of process parameters (Figure 11 and Figure 12).

Figure 11 showcases a remelted surface for process parameters that typically lead to a significant reduction in surface roughness of steels. However, in case of AISI D2 a variety of different surface structures were induced to the surface, so that surface roughness could not be significantly reduced. A small scanning velocity and a large laser beam led to long interactions times, which resulted in pronounced formation of martensite and consequently an increase in micro-roughness (Figure 11d). Additionally, this combination led to pronounced undercuts and track bulging, which results in an increase in mid-wavelength roughness (Figure 11c). Furthermore, the intense thermal stresses induced by the laser polishing process presumably led to plastic deformations and microstructural changes, which result in surface waviness significantly larger than the laser beam diameter (Figure 11a). However, it is specifically surprising that surface roughness was increased for n = 4 passes in comparison to n = 2 passes. Particularly, the combination of a scanning velocity of *v_scan_* = 50 mm/s and Q200 was unsuited for laser polishing in terms of achieving a low surface roughness. In comparison to Figure 11, Figure 12 demonstrates that a significant increase in scanning velocity helps to reduce effects from process-inherent structure formation in all regions (I–III) such a martensite needles (Figure 12d), track bulging (Figure 12c) and waviness (Figure 12a). Consequently, a small process window was identified in which surface roughness could be reduced to approx. *Ra_min_* = 0.173 ± 0.026 µm. However, it was again surprising that the lowest roughness was achieved for n = 2 passes and not for n = 4 passes.

**Figure 13:**
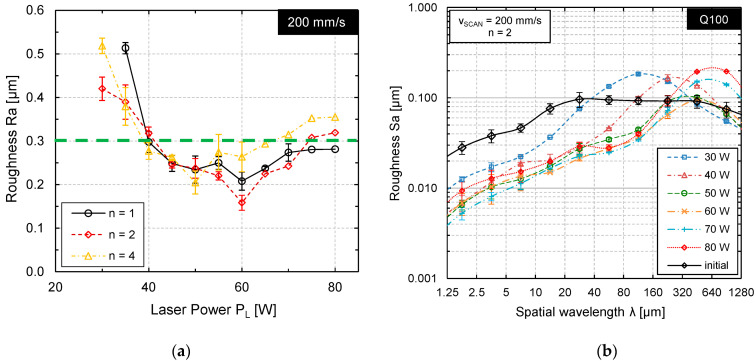
(**a**) Roughness Ra as function of laser power at *v_scan_* = 200 mm/s after n = 1, 2, and 4 passes; (**b**) Sa-spectrum for six different laser powers at *v_scan_* = 200 mm/s after n = 2 passes (Q100, *v_scan_* = 200 mm/s, *dy* = 20 µm).

Based on these results for Q200, a smaller, quadratic laser beam Q100 was investigated to further reduce melt pool volume, heat input, and interaction time. Analogous to the investigation for Q200, the local roughness minimum was determined as a function of laser power for the scanning velocities *v_scan_* = 50, 100 and 200 mm/s as well as for n = 1–4 passes. The results of stylus surface roughness measurements are shown in Figure 13a, while a selected Sa-spectrum for Q100, *v_scan_* = 200 mm/s and n = 2 passes is shown in Figure 13b. For all investigated sets of process parameters, the local minimum for surface roughness was found after multiple processing. In this case, a local roughness minimum of *Ra_min_* = 0.163 ± 0.018 µm was achieved at *P_L_* = 60 W and n = 2 passes (Figure 13).

**Figure 14:**
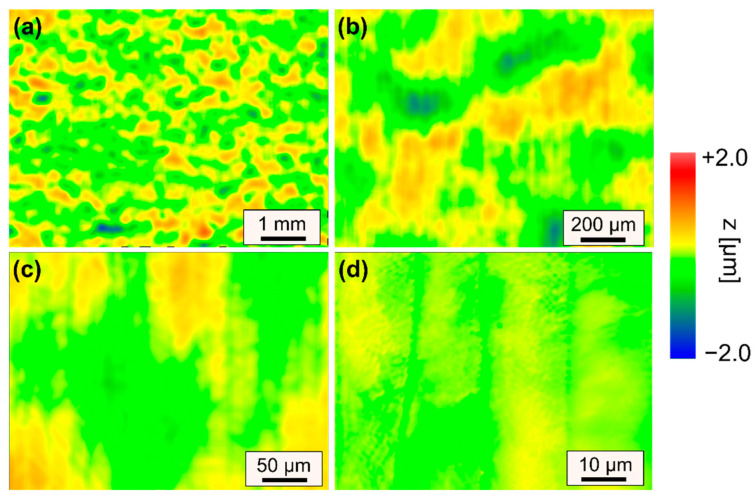
WLI measurements of surface topography for increasing magnifications (**a**–**d**) after laser remelting using Q100 and *v_scan_* = 200 mm/s leading to smallest surface roughness (Q100, *v_scan_* = 200 mm/s, *dy* = 20 µm, *P_L_* = 60 W, n = 2).

The corresponding surface topographies for the minimal surface roughness are shown in Figure 14. It is noticeable that no set of process parameters lead to a reduction of surface roughness for spatial wavelengths longer than 160 µm. The best result was to avoid an increase in macro-roughness and waviness for a laser polished surface.

Overall, for no combination of process parameters hot-cracking or holes were observed after laser polishing. For both laser focus dimensions a tendency towards smaller macro and micro roughness was observed for the highest scanning velocity. It is further noticeable that the micro roughness for spatial wavelengths *λ* < 20 µm at a scanning velocity of *v_scan_* = 200 mm/s is within the same standard deviation for both focus sizes. In the spatial wavelength intervals of *λ* = 20–80 and *λ* = 320–2560 µm, however, significantly larger values were measured for Q200 than for the smaller Q100 laser beam diameter. Based on the spectral roughness analysis, a critical local wavelength of *λ* < 160 µm, up to which the initial roughness could be reduced, was identified for all sets or process parameters. A local maximum in the roughness spectra is typically found in the wavelength range *λ* = 160 to 640 µm for the smaller laser focus and in the range *λ* = 320–1280 µm for the larger laser focus.

### 3.3. Microstructure

**Figure 15:**
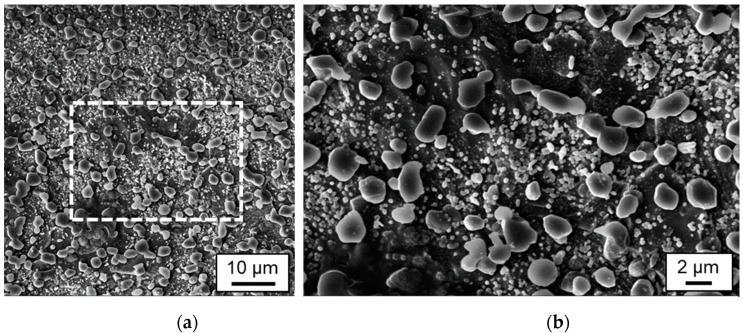
SEM images of microstructure of the initial bulk material in (**a**) smaller and (**b**) larger magnification.

A special feature of the powder-metallurgically melted material 1.2379+ is the small size, the basically spherical or ellipsoidal geometry and the almost statistical distribution of primary carbides in the surrounding iron matrix. In the initial microstructure, the chromium carbides are observed mainly at the grain boundaries, with their size generally being between 1 µm and 3 µm (Figure 15).

**Figure 16:**
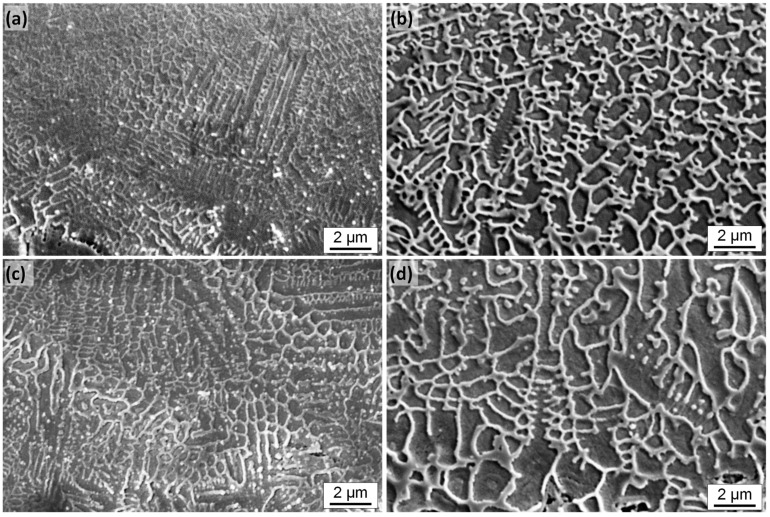
SEM images of metallographically prepared cross-section for single track and surfaces, respectivley, after laser remelting using specific sets of process parameters A–D (Table 3). The set of process parameter A–D corresponds to the letter shown in each images, e.g., (**a**) displays a cross-section for set of process parameters A, (**b**) for set of process parameters B, (**c**) for set of process parameters C, and (**d**) for set of process parameters D.

After laser remelting, the original primary carbides from the initial bulk material are completely dissolved in a new primarily dendritic microstructure. The higher the scanning velocity, the higher the quenching rate and the finer the dendritic microstructure, as is evidenced in the microstructure of laser remelted/polished specimen (Figure 16a,b). In general, for smaller laser beam diameter and larger scanning velocity, a more homogeneous and finer dendritic or cellular structure of presumably austenite and martensite was observed (Figure 16a,c). The darker areas (presumably retained austenite + martensite) are the majority phase, while the brighter areas (presumably eutectic mixture of austenite, martensite, and precipitated carbides) are the minority phase [42]. Typically, in laser remelting, rapid solidification results in austenitic dendrites being formed directly from the liquid phase of the melt pool. During this process, only part of the austenite is transformed into martensite due to high thermal gradients.

Figure 16c shows the microstructure after area laser polishing in comparison to a remelted single track (Figure 16a). It is noticeable here that the microstructure for the areal polishing, i.e., overlapping remelting tracks, is visibly coarser than that of a single track. On the one hand, this is probably due to a heat accumulation caused by the preceding laser processing and, on the other hand, to a subsequent heat treatment caused by subsequent remelting tracks. In both cases, however, the microstructure is significantly finer than in the case of a single track with a larger laser beam diameter Q200 and a lower scanning velocity (Figure 16b). A similar difference was also observed between the microstructure for a single track and areal, i.e., overlapping laser processing for Q200 and *v_scan_* = 50 mm/s (Figure 16d).

**Figure 17:**
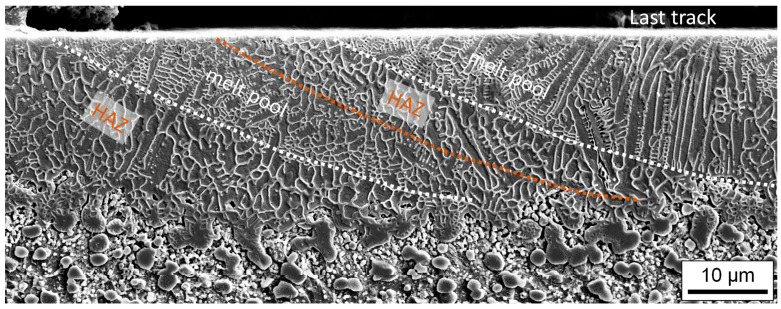
Representative SEM image of metallographically prepared cross-section for set of process parameter D.

Figure 17 shows a metallographically prepared cross-section perpendicular to the scan direction through the last laser remelted track. This last remelted track has a dendritic solidification structure, with the dendrites running in particular perpendicular to the solidification front from bottom to top in the direction of the center of the melt pool surface, i.e., approximately in the direction of the highest temperature gradient. In addition to the primary dendrites, dendrite arms, i.e., secondary dendrites, are also clearly visible. To the left of the last laser remelted track, there is already remelted material from previously remelted tracks. Thus, in addition to the remelted material, a zone is also recognizable in which the material was heat-treated again after remelting by the subsequent remelting track. This heat affected zone (HAZ) can be identified in particular by the fact that almost no secondary dendrites are recognizable. The subsequent heat treatment probably austenitized this area and reduced the amount of martensite in this region.

**Figure 18:**
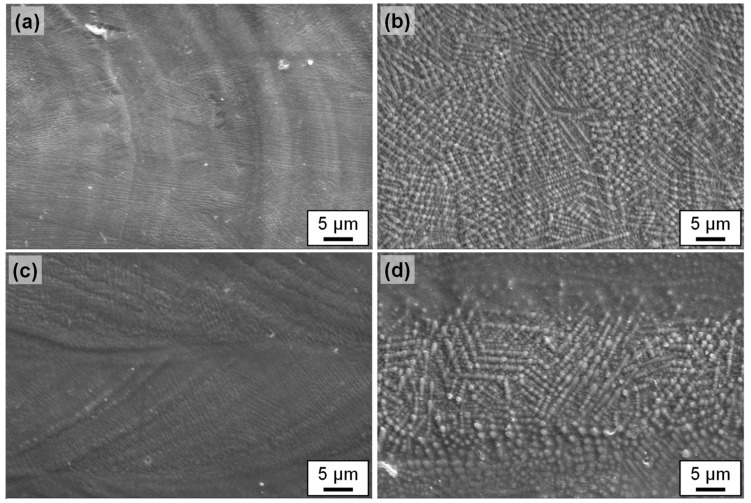
SEM images for single track and surfaces, respectively, after laser remelting for the sets of process parameters A–D (Table 3). The set of process parameter A–D corresponds to the letter shown in each images, e.g., (**a**) displays an image of surface topography for the set of process parameters A, (**b**) for set of process parameters B, (**c**) for set of process parameters C, and (**d**) for set of process parameters D.

Figure 18 shows that the micro-roughness at the surface of the remelted track in particular is probably due to the significantly coarser microstructure for longer laser-material interaction times. (Q200 und *v_scan_* = 50 mm/s: *t_int_* = 4 ms und Q100 und *v_scan_* = 200 mm/s: *t_int_* = 0.5 ms). Figure 18b,d show a pronounced microstructure on the surface, particularly for Q200, which is probably near-surface martensite and significantly increases the microroughness.

In addition, Figure 18d shows a distinctive feature of two-dimensional laser polishing due to overlapping individual tracks. As already explained for Figure 17, heat treatment by subsequent remelting tracks leads to a change in the microstructure, which presumably also results in a reduction of the martensite content. This is also visible on the surface in the form of reduced micro-roughness due to presumably less martensite near the surface. This alternating micro-roughness creates the macroscopic visual stripe-like effect on the surface (Figure 5).

### 3.4. Hardness Measurements

**Figure 19:**
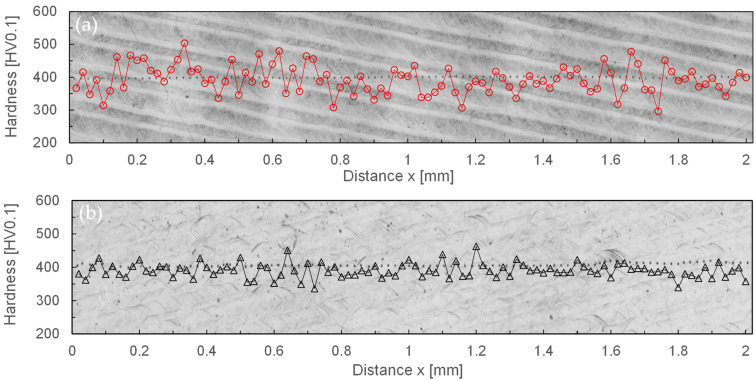
Representative microhardness measurements along lines with 100 measuring points on 2 mm length for laser remelted areas on 1.2379+; (**a**) Q200, *dy* = 40 µm, *P_L_* = 70 W, *v_scan_* = 50 mm/s, and (**b**) Q100, *dy* = 20 µm, *P_L_* = 60 W, *v_scan_* = 200 mm/s.

The initial Vickers hardness H0 of the initial surface was determined, and a micro-hardness of 420 (±210) HV0.1 was determined. The standard deviation on this measurement was very large, depending on whether chromium carbides were completely or partially hit during the measurement. The maximum micro-hardness on the initial surface was approximately 827 HV0.1 (chromium carbides), while the minimum hardness was only 101 HV0.1 (iron matrix). Figure 19 shows representative micro hardness measurements on laser-polished surfaces for the process parameter sets D and C, i.e., for different laser beam dimensions and scanning velocities. For each set of process parameters, 100 micro hardness measurements were evaluated on three different samples.

The average surface hardness in both cases is 390 HV0.1, but the standard deviation in the case of process parameter set D is 46 HV0.1, while the standard deviation for parameter set C is less than half that, at 21 HV0.1. The comparison between Figure 19a,b show that greater local deviations can be seen for process parameter set D and that the hardness measurements as a whole range between the extreme values 296 HV0.1 and 504 HV0.1. For process parameter set C, on the other hand, the minimum hardness value is 336 HV0.1 and the maximum hardness value 463 HV0.1. However, contrary to a corresponding assumption, the measurements did not show a clear correlation of the hardness profile to the dark or light stripes. Further exemplary microhardness measurements also showed that by increasing the number of passes from 1 to 4, no change was observed in the average surface hardness or the standard deviation of hardness for the process parameter sets C and D investigated.

## 4. Discussion

### 4.1. Characteristic Surface Features

#### 4.1.1. Stripes after Laser Processing and Martensite Formation

Massive formation of martensite needles was observed during laser remelting of AISI D2 particularly for small scanning velocities. This is a direct consequence of dissolving the chromium carbides in the melt pool and formation of oversaturated martensite in the resolidified material. The stripes visible on the laser polished surface (Figure 5) are a result of the meandering scanning strategy. Specifically, the formation of undercuts, track bulging and a back tempering effect of subsequent remelting tracks lead to a characteristic appearance of the surface. Specifically, by an increase in scanning velocity and decrease of laser beam dimensions and track offset a more homogenous surface appearance can be created. Stripes next to the laser remelted track (lighter areas in Figure 5) are remelted and heat affected by the subsequent laser track. The reduction of near-surface structures, usually dominated by martensite formation, indicates a significant reduction in the carbon content in these areas. This is supported by the finding that carbon typically diffuses significantly into the melt pool from the peripheral areas of the melt pool (HAZ) due to a higher solubility [9]. A higher carbon content in the melt pool leads typically to an increased martensite formation. This effect is particularly pronounced for large melt pools (large contact area between solid and liquid material) and for low scanning velocities, since interaction time and thus diffusion lengths are largest [43]. However, the precision of the used methods and instrument was not sufficient to clearly identify this difference in carbon content between these two, periodically alternating surface areas. More sophisticated methods such as GDOES were not available for this study. In any case, the heat treatment results in a significantly different microstructure, i.e., larger dimensions of the dendrites, etc., and reduced formation of martensite. The larger dimensions of the microstructure results from smaller temperature gradients in the heat affected zone in comparison to the former melt pool.

#### 4.1.2. Ripple Formation

Intense ripple formation was observed for all sets of process parameters after n = 1 pass. Regardless of the processing parameters (laser power, scanning velocity, track offset), the number of ripples was almost the same. Changes in scanning velocity, laser power and track offset, just resulted in slight changes of the ripple height, which was almost impossible to detect for small scanning velocities since martensite formation dominated the micro-roughness. Since the number of ripples remained almost the same, it is assumed that each chromium carbide might be a source for ripple formation [40]. After the spherical chromium carbides were dissolved in the iron matrix, the number of ripples significantly decreased in the second process step. Furthermore, some ripples created in the first remelting step have such a large height, so that they could not be completely levelled by further passes. Thus, a wavy orange-peel-like surface topography remains specifically in the mid-, and long wavelength intervals of the Sa-spectrum. The mechanism for ripple formation is thought to be similar to controlled surface structure generation in laser remelting (WaveShape process, Temmler and Pirch [27]). The key hypothesis is that any change in the melt pool volume (controlled or uncontrolled) leads to formation of surface structures. In this case, chromium carbides would act as material inhomogeneities in the material matrix, which might lead to changes in melting temperature and/or local changes in the absorption of laser radiation at the surface. Furthermore, dissolving of chromium carbides in the melt pool specifically alters the carbon content and might influence Marangoni convection in the melt pool [44]. However, particularly changes in the melt pool volume due to sporadically dissolving chromium carbides in the molten material are thought to be the reason for surface ripple formation. This is supported by the observation that ripple formation is significantly reduced once the primary and secondary carbides are dissolved in the iron matrix after one laser polishing/remelting step.

The reason for the influence of scanning velocity on ripple formation is presumably that carbides of a specific size have a greater influence on a smaller melt pool than on a larger melt pool. However, since the carbides are basically randomly distributed in the initial bulk material, a larger number of carbides are dissolved in the melt pool for larger laser power and smaller scanning velocities. Both effects counteract each other and lead to no clear dependence of ripple height on process parameters. Ukar et al. [28] for example used a significantly larger laser beam diameter of *d_L_* = 2000 µm, larger laser power of *P_L_* = 750 W and a smaller scanning velocity of *v_scan_* = 32 mm/s for laser remelting of 1.2379. Considering a remelt depth of more approx. 150 µm and 2 mm diameter of the laser beam, the interaction time (*d_L_*·*v_scan_*^−1^; 1/16 s compared to 1/2000 s) and, thus, melt duration were significantly longer and no significant surface rippling was reported by the authors. Additionally, larger laser beam diameter led to longer-waved ripples, since the solidification front has larger geometrical dimensions, which lead to a more pronounced extent of surface ripples [21]. Irrespective of width and length of a ripple, ripple height should directly correlate to the relative change in melt pool volume change (Temmler et al. [45]). However, there was no clear correlation found, which showed that ripple height increases as function of scanning velocity as proposed by Nüsser et al. [21].

In sum, important influences on ripple formation are material inhomogeneities, which lead to changes of the melt pool volume and ultimately lead to ripple formation. Due to dissolving of primary and secondary carbides in the first pass, ripple formation is significantly reduced in subsequent passes, but an orange-peel-like waviness remains on the surface. In any case, form and amount of surface ripples are much more pronounced for cold work steel 1.2379+ (AISI D2) in comparison to tool steel 1.2343 (AISI H11 [9]), tool steel S7 [46], Ti6Al4V [47] or Inconel 718 [48] for example. In general, ripples are typically a direct consequence of continuously changing melting and solidification conditions during remelting and are presumably a direct consequence of microstructural changes (dissolving primary and secondary carbides in the iron matrix).

#### 4.1.3. Undercuts

Undercuts were formed for almost every set of process parameters. An exception was observed for a laser power close to the melting threshold at which only a very shallow surface boundary layer, presumably << 5 µm, was remelted. According to Nüsser et al. [21] the formation of undercuts might be a result of an impeded thermal expansion of the material in the area of the heat-affected zone of a remelted track. Here, the generated stress reaches the yield point of the material and initiates plastic deformation, which remains as an undercut in the surface topography on both sides of the track. Additionally, Kiedrowski [49] hypothesized that due to a small angle of inclination of the melt front at high scanning velocities, the molten material slides down at the melting front, because of the acting capillary forces. The inclination angle is the angle between the original surface and the melt front. The contact angle is the angle between the melt pool surface and the melt front. In case of a flat melt pool surface, the angle of inclination and contact angle coincide. The contact angle of the melt pool surface to the melt or solidification front is not constant but results from the solution for the free boundary value problem for surface remelting using laser radiation (Temmler and Pirch [27]). It depends therefore not only on the material properties but on the entire dynamics of the laser remelting process. The reason for this is presumably that the contact angle is significantly smaller than the wetting angle. As the three-phase line slides, the contact angle increases continuously, presumably until the wetting angle is reached. The sliding of the molten material in the transition area from the melt front to the solidification front is thought to lead to formation of undercuts that can be seen on both sides of laser remelted track. Additionally, Temmler et al. [45] found that the shape of a melt pool surface in laser remelting and specifically the inclination angle along the three-phase line is dominated by the normal forces of the Marangoni flow. Particularly, in the transition zone between melt front and solidification front, the largest normal forces along the three-phase line were found. As a result, the surface tension gradient perpendicular to the scanning direction is significantly larger than at the rest of the three-phase line. This was hypothesized to be the physical reason for the formation of undercuts during laser remelting [45]. In sum, undercuts are presumably a consequence of capillary forces [49], thermo-capillary forces [27], and thermo-mechanical forces [21]. In the case of AISI D2, the acting forces cannot be separated from each other and lead, among other things, to the fact that clearly visible undercuts appear for almost all combinations of process parameters examined.

### 4.2. Laser Polishing Power and Surface Roughness Evolution Topography

The first step in laser polishing is usually to determine the laser power at which surface roughness is reduced the most (laser polishing power). The largest reduction in surface roughness is typically achieved using large laser beam diameter and small scanning velocities [4]. Long interaction times of laser radiation and material typically lead to long melt durations. This leaves the most time for capillary forces to level the molten surface [26], and has the effect that capillary surface waves are effectively damped [50]. Therefore, surface roughness up to spatial wavelengths of approximately four times the laser beam diameter are typically effectively reduced [51]. However, the specific results for the smallest scanning velocity and the largest laser beam diameter showed that particularly the macro-roughness and waviness were significantly increased by laser remelting. Therefore, the standard approach for laser polishing does not seem to work for tool steel AISI D2. This is not only the case for the waviness but also for meso-roughness and micro-roughness of the resulting surface. Under consideration of the specific process- and material induced surface features such as martensite needles, ripples, track bulging, and undercuts, each significant increase in a spatial surface roughness interval could be addressed to one specific surface feature. Untypically for many metals, the tool steel AISI D2 shows almost all kind of process induced surface structures, which eventually increase the surface roughness. Nonetheless, the formation of these surface features can effectively be suppressed, if small laser beam dimensions, high scanning velocities and small track offsets are used. However, this has the main disadvantage that generally only surface features with spatial wavelengths smaller than approx. 100 µm can be effectively smoothed by laser polishing (starting Ra_ini_ = 0.33 ± 0.026 µm). Compared to other steels, e.g., 1.2343 [52], 420 stainless steel [53], Ti6Al4V [47] or 1.4571 [49], this critical spatial wavelength is smaller for AISI D2, since for, e.g., tool steel H11 typically spatial wavelengths up to 1 mm are effectively smoothed by a laser beam diameter of approx. *d_L_* = 250 µm [1]. Furthermore, an increase of the passes does not necessarily lead to a smaller surface roughness. The second laser polishing pass effectively and significantly reduces remnant ripples from the first pass and produces far less and smaller surface ripples, which lead in sum to a further reduced surface roughness. However, a further increase of the laser polishing passes to n = 4 resulted in a higher surface roughness in almost all cases (compared to surface roughness after n = 2 passes). The reason for this presumably lies in heat accumulation in the laser processing area, which might lead to unfavorable thermal gradients among others leading to a coarser microstructure, which might affect the resulting surface roughness. This indicates further that not only laser polishing parameters and number of remelting steps but also the timing between laser polishing steps might be crucial to achieve a low surface roughness on AISI D2.

Overall, the results show that high scanning velocity, small laser beam diameters and two laser polishing steps are beneficial to reduce surface roughness. However, if the waviness should be reduced no set of process parameter could be found to further reduce surface roughness below Ra_min_ = 0.163 ± 0.018 µm. Nonetheless, in comparison to, e.g., Ukar et al. [28], a significantly lower surface roughness was achieved in this study.

### 4.3. Microstructure

#### 4.3.1. Hot Cracking and Pores

No hot-cracking or pores were observed. Since hot-cracking is typically a problem in laser remelting of D2, e.g., Ukar et al. [28] found hot-cracking for low intensities and small scanning velocities, comparatively small cooling rates and long interaction times. Maybe the main difference lies in the Gaussian laser beam intensity distribution of CO_2_ laser beam in comparison to a square top heat beam in this study. However, in general, it seems that hot-cracking can be avoided by a correct choice of suitable process parameters. Regarding pores and gas porosity, Pleterski et al. [54] assumed that trapped impurities, most likely resulting from overlapping welding tracks (oxides on previously remelted track, formed due to insufficient gas shielding or turbulences) or from a poorly cleaned surface (oxides, lubricants) are the main reason for the occurrence of pores and porosity. These pores and large carbides are typically the main reason for (hot-) cracking of the remelted source. Since laser remelting occurs in a shielding gas atmosphere, the surface was cleaned in an ultrasonic bath, a homogeneous, quadratic top-hat intensity distribution was used and carbides were largely dissolved in the iron matrix, no cracks were observed during laser remelting.

#### 4.3.2. Microstructure, Cooling Rate and Hardness

Primary carbides that are widely present in the initial bulk material are completely dissolved in the laser remelted regions. The high cooling rates of the molten material, due to the short interaction time between laser radiation and material (*d_L_·v_scan_*^−1^ << 1s), and the comparatively small melt volume (*V_melt_* << 1 mm^3^) together result in the absence of M_6_C carbides, which is in accordance with, e.g., Arias et al. [55] and others. The darker regions in Figure 16 are presumably, mainly austenitic dendrites with some amount of martensite, while the brighter interdendritic interstices presumably consist of a eutectic system of martensite and small precipitated carbides (M_23_C_6_ and M_7_C_3_) [56]. Typically for laser remelting, rapid solidification results in austenitic dendrites being formed directly from the liquid phase of the melt pool. During this process, only part of the austenite is transformed into martensite due to high thermal gradients. Additionally, a eutectic reaction typically leads to a decomposition of the interdendritic liquid into eutectic carbides and secondary austenite, which contains typically smaller amounts of carbon and other alloy elements compared to the primary austenite of the dendrites [55].

During solidification in laser remelting, grains have a strong tendency to grow preferably in the direction of the largest temperature gradient [39]. In a stationary case this would mean that the grain growth direction would be perpendicular to the phase front between solid and liquid material, i.e., from the bottom of the melt pool towards the center of the melt pool near the surface. In a dynamic case, this means for a moving heat surface such as a laser beam on a metallic surface such as in laser remelting, a high temperature gradient is apparent in the direction opposite to the main scan direction. Therefore, preferred growth directions are preferably bottom to top and anti-parallel to the scan direction as it was observed in Figure 16 and Figure 18. The microstructure within the grains results from the solidification conditions, which might locally change due to local temperature gradients and results into cellular, planar, equiaxed dendritic or columnar dendritic growth depending on the specific conditions at the phase boundary solid/liquid.

Characteristic for laser remelting is that the finer the resulting microstructure of a remelted material, the higher the cooling rate during laser processing [57]. In this context, Temmler et al. [9] came to the following interrelationship between cooling rate Δ*T*/Δ*t* (temperature gradient) and significant process parameters in laser remelting (Equation (2)).

(2) $$\;\frac{\Delta T}{\Delta t}\propto \frac{I}{\sqrt{t_{int}}}\cdot const.\;\mathrm{with}\;I=\frac{P_{L}}{d_{L,E}{}^{2}}\;\mathrm{and}\;t_{int}=\frac{d_{L,E}}{v_{scan}}$$

*t_int_*: interaction time, *I*: laser power per irradiated area (intensity) *const*. is a constant calculated from thermal conductivity, density, absorptivity, and specific heat capacity

For the investigated and compared process parameters Q100 at *v_scan_* = 200 mm/s and Q200 at *v_scan_* = 50 mm/s a short calculation using Equation (2) leads to an approx. 10 times higher cooling rate for the smaller laser beam at higher scanning velocity. This difference in cooling rate and the dependent difference in microstructure is clearly visible in the comparison of, e.g., Figure 16a,c.

A rapid reheating of the previously melted material, e.g., by subsequent remelting tracks in areal laser polishing without exceeding melt temperature, typically leads to an austenitization of the microstructure [58]. The repeated rapid heating of the material possibly results in a homogenization of the austenite composition within the dendrites and the interdendritic interstices. This homogenization of the austenite presumably resulted in a lower amount of martensite in the interdendritic interstices at ambient temperature, which had the visual effect on the surface of showing less micro-roughness due to less martensite near the surface (Figure 6 and Figure 18d). The alternation between the melt zone and heat affected zone (HAZ) with less martensite results in the striped appearance of the laser polished surface (Figure 5a).

By means of laser remelting, the surface hardness was largely homogenized to 390 HV0.1 over the entire laser-polished area. Presumably, the larger deviation of the measured values for the process parameter set Q200, *v_scan_* = 50 mm/s, *dy* = 40 µm is due on the one hand to the larger track offset and on the other hand to the larger area of the heat-affected zone. Due to the subsequent laser remelting tracks, a subsequent heat treatment takes place, which is temporally the longer and locally the more extended, the larger the laser beam dimensions and the smaller the scanning velocity. This assumption was basically confirmed by the larger standard deviation of the surface hardness. However, no clear correlation between the differently bright areas on the laser polished surface could be established experimentally. Overall, however, it can be stated that although the microstructures for process parameter sets C and D differ significantly, this has no influence on the resulting surface hardness after laser polishing. A further increase in surface hardness up to 464 HV0.1 could, however, be achieved, e.g., by subsequent laser micro polishing (LµP) with pulsed laser radiation [40].

## 5. Conclusions

In this work we investigated cw laser polishing of tool steel 1.2379 (AISI D2) for two different laser beam sizes (square, tophat-like intensity distribution), three different scanning velocities and up to four remelting/polishing steps. We found that the best results (lowest surface roughness) were achieved for the smaller laser beam dimensions (Q100) and the highest scanning velocity (*v_scan_* = 200 mm/s). Additionally, some noteworthy insights of the experimental study on LP of 1.2379 are as follows:

- The standard approach for laser polishing (large laser beam, small scanning velocity, many passes) does not seem to work for tool steel AISI D2. A significant reduction of surface roughness from Ra_ini_ = 0.33 ± 0.026 µm to Ra_min_ = 0.163 ± 0.018 µm was only possible for the smallest interaction time (Q100, *v_scan_* = 200 mm/s), which also resulted in the finest microstructure.
- While increasing the number of passes to n = 2 leads to a lower surface roughness (remelting and reduction of surface ripples), a further increase to n = 4 is not beneficial.
- Process inherent surface structure formation such as martensite formation, formation of undercuts, track bulging significantly influences the resulting surface topography and roughness.
- Near-surface chromium carbides are completely dissolved in the regions where the material was remelted.
- The HAZ showed less near-surface martensite, which resulted in a reduced micro-roughness. Reaustenitization in the HAZ lead to disappearance of secondary dendrites of the dendritic solidification micro-structure.
- A stripe-like structure was visible on the surface after laser polishing, which resulted from alternating differently heat-affected zones.
- The resulting average surface hardness was 390 HV0.1 and the same irrespective of the laser polishing parameters and the number of remeltings; only the standard deviation of surface hardness was more than doubled from 21 HV0.1 to 46 HV0.1 for larger laser beam, smaller scanning velocity and larger track offset.

Overall, the material 1.2379+ poses several significant challenges for the laser polishing by remelting process. To minimize process inherent surface structure formation such as martensite formation, undercuts or orange-peel like waviness, short interactions times should be preferred over longer ones. This is approach is contrary to traditional laser polishing approaches using larger laser beam dimensions and smaller scanning velocities.

## Figures and Tables

**Table 1:** Tabular overview of chemical composition for AISI D2 (in wt.%).

Material/Element ^1^	C	Si	Cr	Mo	Mn	V	Fe
AISI D2	1.56	0.4	11.86	0.83	0.38	0.84	Bal.
Dev.	±0.1	±0.1	±0.45	±0.2	±0.1	±0.1	-

^1^ Based on supplier information.

**Table 2:** Tabular overview of process parameters and range of investigation.

Process Parameter	Q100	Q200
Process steps	1, 2, 4	1, 2, 4
Repetition rate *f_rep_*	cw	cw
Laser beam diameter *d_L_* [µm]	100	200
Scanning velocity *v_scan_* [mm/s]	50, 100, 200	50, 100, 200
Laser power *P_L_* [W]	20–80	40–160
Track offset *dy* [µm]	20	40
Passes n	1, 2, 4	1, 2, 4
Processing angle α [°]	0/90	0/90
Shielding gas	Ar + O_2_	Ar + O_2_
Residual oxygen *c*(*O*_2_) [ppm]	1000	1000

**Table 3:** Sets of chosen process parameters for microstructural (A–D) and micro-hardness analysis (C, D).

Set of Parameters	*d_L_*	*v_scan_* [mm/s]	*P_L_*	*dy*	n	α
[Physcial Units]	[µm]	[mm/s]	[W]	[µm]	n.a.	[°]
A	100	200	60	1000	1	0
B	200	50	70	1000	1	0
C	100	200	60	20	1	0
D	200	50	70	40	1	0

## Data Availability

The data presented in this study are available on reasonable request from the corresponding author.
